# CoP/Co_2_P heterostructures embedded in biomass-derived carbons as efficient electrocatalysts for hydrogen and oxygen evolution reactions

**DOI:** 10.1039/d6ra05091c

**Published:** 2026-08-07

**Authors:** Abdourahmane Dieng, Ahmed Subrati, Zahra Hagheh-Kavousi, Ndeye M. Ndiaye, Yaovi Holade, Eduart Gutierrez-Pineda, Sergio E. Moya, Balla D. Ngom

**Affiliations:** a Laboratoire de Photonique Quantique d’Energie & NanoFabrication (LPQEN), Faculté des Sciences et Techniques, Département de Physique, Université Cheikh Anta Diop de Dakar BP5005 Dakar-Fann Dakar Senegal bdngom@gmail.com +221 77 441 66 44; b Center for Cooperative Research in Biomaterials (CIC biomaGUNE), Basque Research and Technology Alliance (BRTA) San Sebastian 20014 Spain; c Institut Européen des Membranes, IEM, UMR, 5635, Univ. Montpellier, ENSCM, CNRS 34090 Montpellier France

## Abstract

The development of efficient and cost-effective bifunctional electrocatalysts for the hydrogen evolution reaction (HER) and oxygen evolution reaction (OER) is critical for sustainable energy conversion. In this study, cobalt phosphide-based nanocomposites were synthesized *via* a simple hydrothermal and phosphidation process, using biomass-derived porous carbon as a sustainable support. Peanut-shell extract-derived carbon (BC) and activated carbon (AC) were used as carbon supports to obtain the CoP/Co_2_P@BC and CoP/Co_2_P@BC@AC composites. Two types of novel heterostructures were fabricated: CoP/Co_2_P@BC and CoP/Co_2_P@BC@AC. However, the optimized CoP/Co_2_P@BC@AC catalyst demonstrated outstanding bifunctional electrocatalytic performance, requiring overpotentials of only −227 mV for the HER and 303 mV for the OER to achieve a current density of 10 mA cm^−2^. Furthermore, it exhibited Tafel slopes of 121 mV dec^−1^ and 101 mV dec^−1^ for HER and OER, respectively, along with excellent long-term durability, confirming its high efficiency for overall water splitting under alkaline conditions. The outstanding catalytic behavior was mainly ascribed to the amorphous framework, large active surface area, and enhanced charge-transfer capability of the catalyst. These findings underscore the potential of biomass-derived carbon as a platform for fabricating stable, low-cost, and efficient bifunctional electrocatalysts for water-splitting applications, fostering environmentally benign energy technologies.

## Abstract 2

The development of efficient and cost-effective bifunctional electrocatalysts for the hydrogen evolution reaction (HER) and oxygen evolution reaction (OER) is critical for sustainable energy conversion.

## Graphical abstract

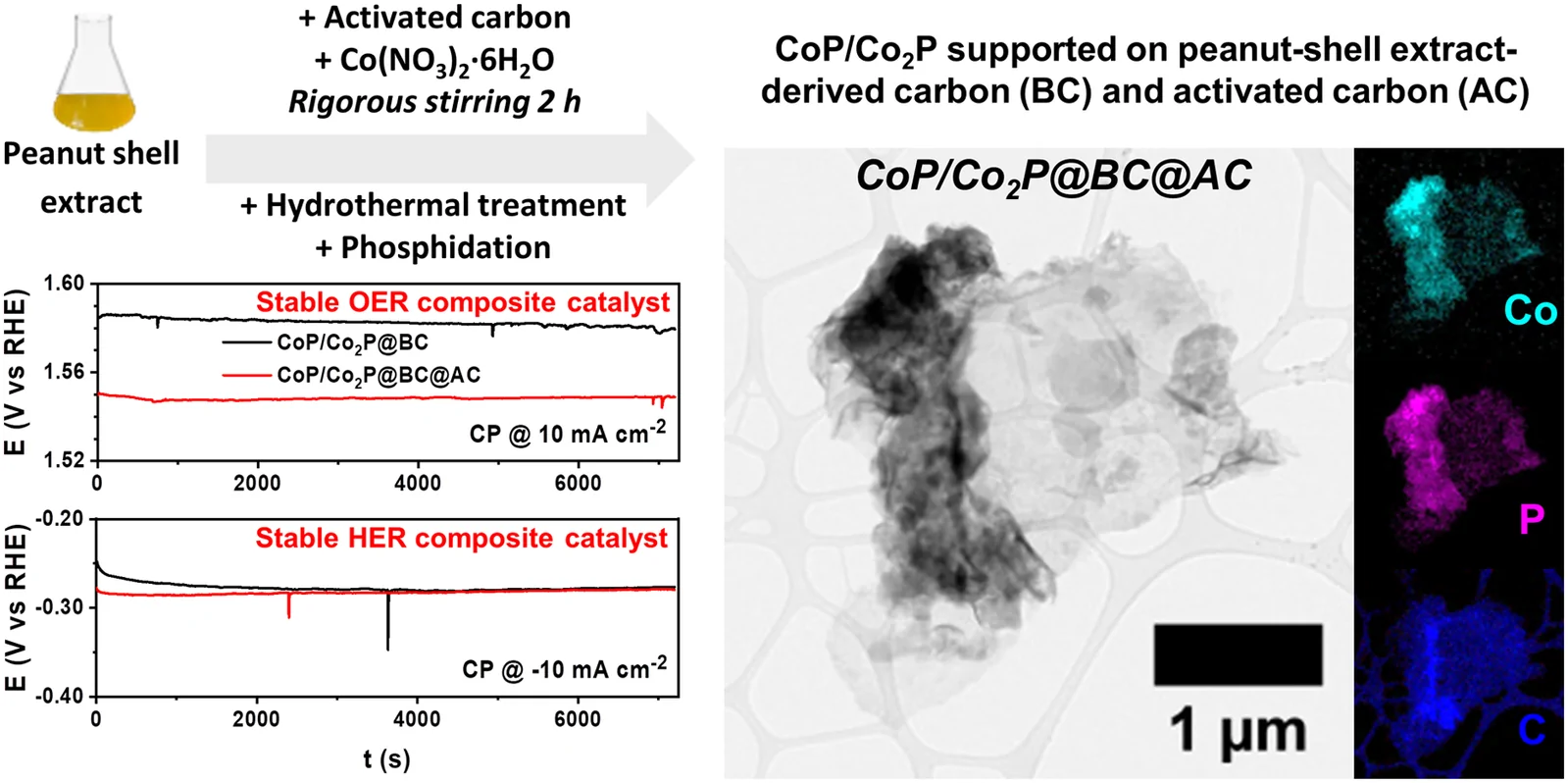

## 1 Introduction

The application of bifunctional electrocatalysts to water splitting is a recent trend that minimizes the cost of hydrogen production by simplifying electrolysis and saving resources.^1^ Electrochemical water splitting involves two fundamental reactions: the hydrogen evolution reaction (HER) at the cathode and the oxygen evolution reaction (OER) at the anode. These reactions can occur in acidic, alkaline, or neutral electrolytes under ambient or high-temperature conditions.^2^

On the material choice aspect, platinum (Pt) is the best choice for HER catalysts because of its low overpotential and exceptional activity.^3–6^ In addition, Noble metals, and in particular the Pt–Ru–Ir series, have been considered ideal electrocatalysts for the water electrolysis by reducing the overpotential during the electrocatalytic hydrogen evolution reaction (HER) and oxygen evolution reaction (OER), but the high cost and low earth abundance of these materials have limited their use in large-scale H_2_ production technologies.^7–9^

However, low-cost water electrolysis systems can be fabricated using bifunctional electrocatalysts by reducing the costs of precursors and material synthesis, purification, characterization, *etc.*^1^ Currently, transition metal phosphides (TMPs), particularly CoP, have garnered attention as probable substitutes for Pt owing to their favorable electrical conductivity, exceptional chemical stability, and electronic structure.^10,11^

However, cost-effective and high-efficiency non-precious-metal bifunctional electrocatalysts for catalyzing the HER and OER, including phosphides,^12^ sulfides,^13^ carbides,^14^ and oxides^15,16^ are needed. Thus, cobalt phosphide (CoP) has emerged as a catalyst of high potential owing to its high catalytic activity and durability in water splitting.^17^

CoP-based materials have been used exclusively for OER. The use of single material for both HER and OER would be beneficial as it would reduce the production cost of the electrodeposition and enhance the stability and simplicity of the system.^18^

Cobalt phosphide (CoP) was selected as the active material in this work owing to its metallic-like conductivity and versatile Co^2+^/Co^3+^ redox behavior, which enables fast, reversible faradaic reactions, and lowers internal resistance compared with cobalt oxides or hydroxides.^19^ Nevertheless, single-phase CoP electrodes remain prone to interfacial instability and active-site loss upon prolonged cycling, limiting their long-term structural stability and practical application.^20^

To overcome these limitations, we constructed a CoP/Co_2_P heterostructure on biomass extract (CoP/Co_2_P@BC) and then combined it with activated carbon to obtain the ternary composite CoP/Co_2_P@BC@AC. The CoP/Co_2_P interface offers abundant active sites that can promote charge transfer, while the conductive, biomass carbon helps disperse the nanoparticles evenly and improves electrolyte access. Adding activated carbon further boosts conductivity, enlarges the accessible surface area, and speeds up ion transport. Together, these features should improve charge-transfer kinetics, ion diffusion, structural stability, and the overall electrochemical performance of the composite.^19^

Consequently, the preparation of electrocatalytic materials with high catalytic activity using more economical carbon sources remains a major challenge. However, biomass has been considered as a precursor of a carbon matrix with various structures.^21,22^

At present, corn straw, peanut shell, luffa sponge, cork bark are commonly used as precursors for the synthesis of various graphene-like carbon materials.^23^ Biomass-based catalysts have several advantages: firstly, the high quantity and availability guarantee the feasibility of industrial production of biomass-based catalysts.^24,25^ Secondly, the diversity and different morphologies demonstrate great flexibility during the biomass carbonization process with controllable shape, surface and porosity.^26^ Thirdly, the biomass-based route to catalyst synthesis is environmentally friendly and has enormous potential for further research in many areas such as catalysts, materials and biochemistry.^27^ Indeed, biomass can not only be a source of carbon,^28,29^ but can also play a crucial role in catalyzing chemical reactions.^30^

For the same purpose, Ngom *et al.* have also successfully synthesized vanadium pentoxide carbon composite (V_2_O_5_@C) derived from the natural *Hibiscus sabdariffa* (HS) family using a green solvothermal process. They concluded that a high amount of carbon from the HS extract was incorporated into the V_2_O_5_ matrix, which improved the electrochemical performances.^31,32^

Consequently, it is imperative to develop low-cost, high-performance electrocatalysts for the large-scale application of electrochemical energy devices. In this study, we have established a facile and inexpensive approach to synthesize heterostructure catalyst materials CoP/Co_2_P@BC and CoP/Co_2_P@BC@AC as bifunctional catalysts *via* a hydrothermal route with peanut shell extract and activated carbon derived from peanut shell as the precursors followed by a phosphidation final step.

The as-prepared CoP/Co_2_P@BC@AC composite demonstrates superior activity and durability for OER, requiring an overpotential of 303 mV at a current density of 10 mA cm^−2^, and long-term stability in an alkaline solution, in contrast to CoP/Co_2_P@BC requiring an overpotential of 326 mV at the same current density.

However, CoP/Co_2_P@BC exhibited remarkable catalytic performance for HER in alkaline media, with a current density of 10 mA cm^−2^ at overpotentials of 250 mV, in contrast to CoP/Co_2_P@BC@AC composite requiring an overpotential of 227 mV at the same current density.

This work brings new ideas for future advances in the construction of bifunctional catalysts based on non-precious, low-cost, efficient metals and graphitic carbon derived from biomass that have both HER and OER efficiencies. Throughout this manuscript, biomass extract and activated carbon are abbreviated as BC and AC, respectively; the resulting composites are accordingly denoted CoP/Co_2_P@BC and CoP/Co_2_P@BC@AC.

## 2 Experimental details

### 2.1. Materials

Reagents Co(NO_3_)_2_·6H_2_O (purity 99%), NaH_2_PO_2_·6H_2_O, (purity 99%) purchased from Sigma-Aldrich were directly used without any purification. Potassium hydroxide (KOH, 99.98% (trace metal basis), Acros Organics). The peanut shell (PS) was collected in Senegal. A nickel foam for battery cathode substrate (bcnf-05m) (0.5 mm *T* × 200 mm *W* × 1 m *L*), surface density: 350 g m^−2^, area 0.2 m^2^ was used as material support and current collector. Ultrapure Ar (grade 5.0, Air Liquide France) was used. The used water was ultrapure with a resistivity of 18.2 MΩ cm at 20 °C and was provided by a Milli-Q Millipore source, deionized water (DI).

### 2.2. Preparation of the samples

#### 2.2.1. Synthesis of activated carbon (AC)

The peanut shells waste (PSW) was collected as raw material and rinsed by soaking the peanut shells in distilled water several times to remove impurities. The sample was then dried in an oven at 60 °C for a sufficiently long period. The dried hulls were then ground to a powder using a blender. To prepare the hydrochar, 10 g of peanut shell powder was mixed with 100 mL of distilled water and the mixture was put in an autoclave (hydrothermal carbonization) at 200 °C for 12 hours. Thus, the product obtained was filtered, and the resulting homogeneous brown gel was dried in an oven at 60 °C. Activated carbon was prepared using a conventional pyrolysis technique in the same experimental conditions reported by Sylla *et al.*^33^ using 5 g of the dried hydrochar mixed with a 4 M solution of KOH. The resulting tab was placed in a mould and dried for 24 hours at 60 °C in an oven before being transferred to a CVD at 750 °C at 5 °C min^−1^ in the presence of a 250 mL min^−1^ flow of Ar gas. The resulting product was soaked in 3 M HCl, then sonicated for 12 hours to remove all traces of unreacted KOH, and rinsed with distilled water to achieve a neutral pH. The sample was dried for further characterization.

#### 2.2.2. Synthesis of CoP/Co_2_P@BC and CoP/Co_2_P@BC@AC composites

**Fig. 1:**
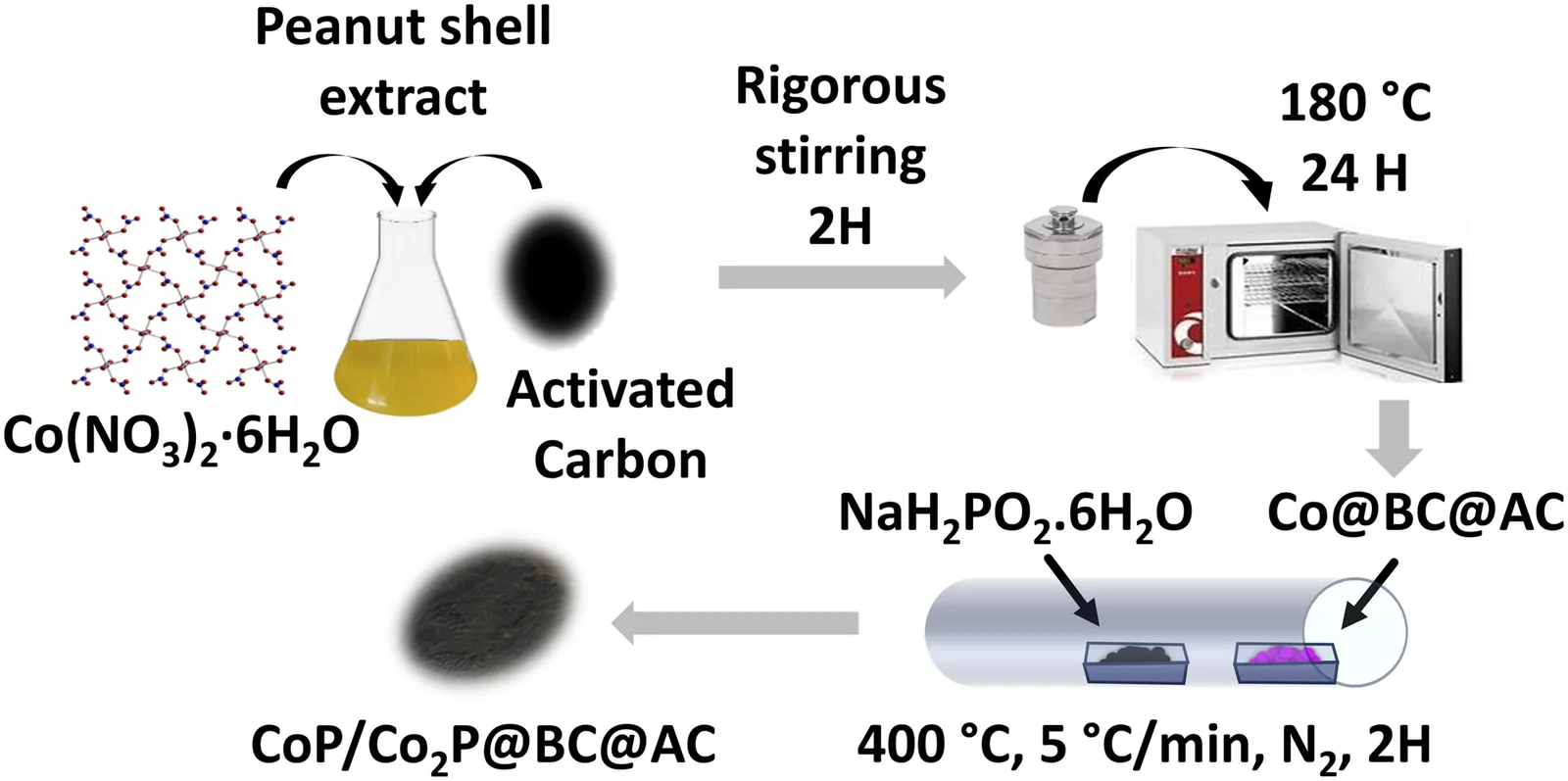
Synthesis of CoP/Co_2_P@BC@AC.

For the composites' preparation, 3 g of the peanut shells powder was added into 150 mL of distilled water, and the resulting mixture was left to stand for 24 h at room temperature to ensure maximum extraction of the bioactive compounds. The resulting mixture was then filtered to remove solid residues of the peanut shell. A clear, yellowish filtrate was obtained and used to synthesize the composite materials. Fig. 1 shows the protocol described for the synthesis.

Two steps were used to synthesize CoP/Co_2_P@BC from the peanut shell: step1-hydrothermal synthesis with dye extraction and followed by step2-phosphorating.

The hydrothermal synthesis was performed using 1 g of Co(NO_3_)_2_·6H_2_O and 30 mL of the above dye obtained from peanut shell extract. This mixture was dissolved under continuous magnetic stirring for 2 hours to homogenize the solution, then transferred into a 50 mL Teflon-lined autoclave and maintained at 180 °C for 24 h. The autoclave was allowed to cool down naturally to room temperature, and the as-obtained product was then recovered, filtered and dried at 60 °C overnight.

For the synthesis of CoP/Co_2_P@BC, 10 mg of the product was placed in a porcelain boat and another porcelain boat containing 1 g NaH_2_PO_2_ was placed at the upstream of the tube furnace. The two alumina boats were calcined at 400 °C for 2 h at a heating rate of 5 °C min^−1^ under a N_2_ flow and then cooled to room temperature naturally.

Similarly, the same process was used to synthesize the CoP/Co_2_P@BC@AC composite by adding activated carbon from peanut shell.

### 2.3. Physical characterization

Infrared spectra were recorded using a Bruker Invenio-X Fourier transform infrared (FTIR) spectrometer in Attenuated Total Reflectance (ATR) mode, covering the wavenumber range of 4000–400 cm^−1^, with a resolution of 2 cm^−1^ and 32 scans per sample. Dried powder samples were directly placed on the diamond crystal without any additional preparation. Raman spectra were obtained using an InVia Reflex confocal Raman microscope (Renishaw), comprising an optical microscope (Leica) with an *XYZ* scanning stage (prior), equipped with a Peltier-cooled front-illuminated 1024 × 512 CCD detector and a 1800 L mm^−1^ diffraction grating. For the experiments, a 532 nm laser excitation source and a 10× objective (numerical aperture, NA = 0.8) were used. Scanning electron microscopy (SEM) was conducted on a JEOL SEM JSM-IT800HL (Tokyo, Japan) equipped with an energy dispersive X-ray spectroscopy (EDS) system, ULTIM EXTREME SEM, with AZTEC software from OXFORD INSTRUMENTS PLC & SUBSIDIARIES (Abingdon, UK). For the High-Resolution Transmission Electron Microscopy (HR-TEM) studies, samples were dispersed in ethanol. Before drop casting on lacey carbon grids (Ted Pella Inc., USA), dispersions were subjected to soft sonication until they became homogeneous. The grids were left to dry under ambient conditions prior to investigation. HR-TEM experiments were conducted on a JEOL JEM-2100F UHR electron microscope (200 kV) equipped with an Energy Dispersive X-ray (EDS) detector (Oxford UltimMax) and two Scanning Transmission Electron Microscopy (STEM) detectors: Bright-Field (BF) and High-Angle Annular Dark-Field (HAADF). XP spectra were recorded using the PHI XPS VersaProbe III energy spectrometer equipped with a monochromatic 1486.6 eV Al-Kα radiation source. A focused X-ray source with X-ray beam size 100 µm, power: 25 W, and e-beam energy: 15 kV was used. Charge neutralization was possible by using a complementary dual beam charge neutralization method. The C 1s peak at 284.8 eV was used as reference to calibrate acquired spectra.

### 2.4. Electrochemical measurements

The half-cell study was carried out in a single-compartment, three-electrode electrochemical cell, consisting of a home-made double-shell glass cell with water circulation at 25 °C, using a VSP-3e potentiostat (BioLogic Science Instruments) controlled by the EC-lab software. The counter and reference electrodes were glassy carbon (6 cm^2^) and a reversible hydrogen electrode (RHE, HydroFlex® Hydrogen Reference Electrode, BioLogic), respectively. The working electrode was made by cutting Ni Foam into an L-shape, measuring 1 cm by 1 cm, with a 0.3 cm by 1 cm section at the top for electrical wiring with gold. The electrolyte consisted of 1 M KOH. The catalytic ink was prepared using ultrasonic mixing in an ice bath (Elmasonic sonication bath, Grosseron, France): first, mixing 400 µL of MQ water with 400 µL of iPrOH and 12.8 mg of Nafion for 20 minutes, then adding 16.0 mg of each studied catalyst material and mixing for another 20 minutes. A 50 µL drop of this ink was applied to each side of the Ni Foam and dried at room temperature. This process resulted in a catalyst loading of 82 µg per cm^2^ per catalyst, matching the ranges used in our synthesis to ensure a fair performance comparison. Electrochemical measurements were performed using cyclic voltammetry (CV), linear sweep voltammetry (LSV), potentiostatic electrochemical impedance spectroscopy (EIS) and chronopotentiometry (CP).

## 3 Results and discussion

### 3.1. FT-IR spectroscopy

**Fig. 2:**
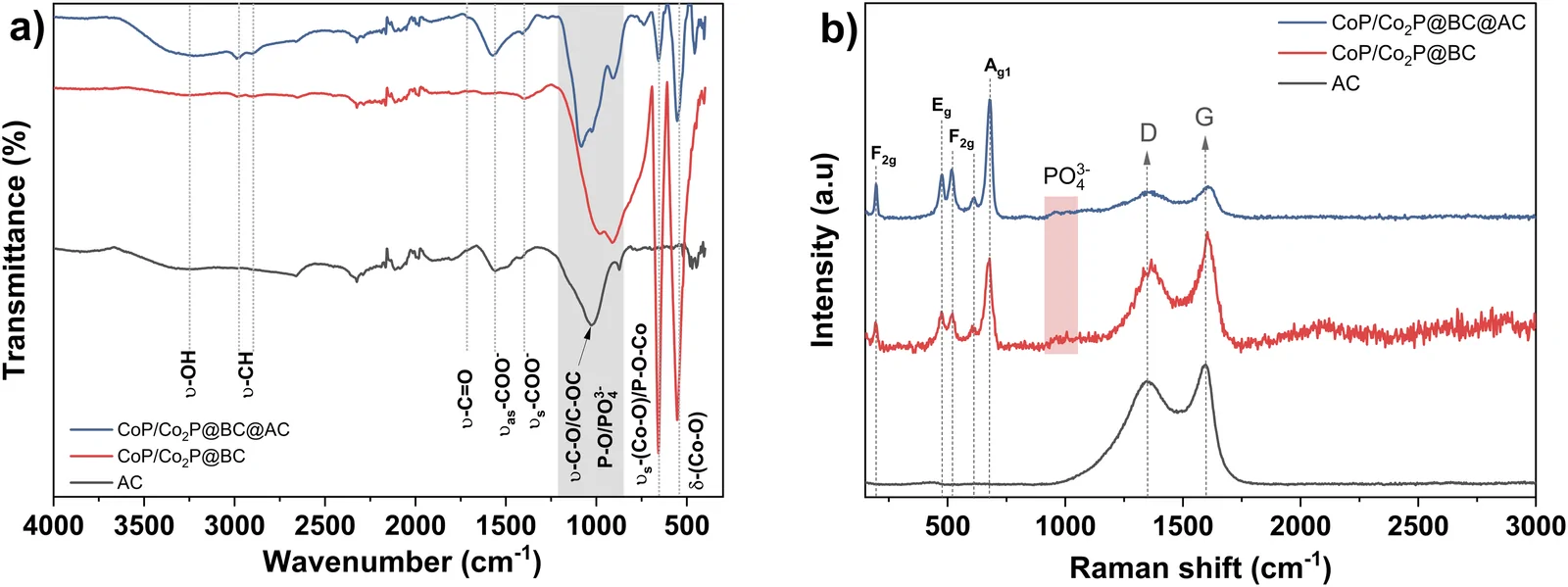
(a) FTIR spectra, and (b) Raman spectra of AC, CoP/Co_2_P@BC, and CoP/Co_2_P@BC@AC.

FTIR spectroscopy was used as a complementary technique to probe the surface oxygenated functionalities and oxidized phosphorus-containing species of AC, CoP/Co_2_P@BC and CoP/Co_2_P@BC@AC (Fig. 2a). All samples display a broad absorption in the 3200–3300 cm^−1^ region, assigned to *ν*(O–H) vibrations of surface hydroxyl groups and adsorbed water. Weaker features between 2900 and 2800 cm^−1^ arise from the symmetric and asymmetric stretching of residual aliphatic C–H groups. The bands in the 1750–1700 cm^−1^ region are associated with *ν*(C=O) vibrations of carboxylic, lactone and related oxygen-containing groups, whereas the features around 1560 and 1400 cm^−1^ are assigned to the asymmetric and symmetric stretching of carboxylate groups, respectively. These functionalities are characteristic of biomass-derived and activated carbons and may provide anchoring sites for cobalt-containing species.

The most relevant phosphorus-related changes occur within the congested 1200–900 cm^−1^ region. Surface-bound phosphate species have previously been associated with an absorption near 1080 cm^−1^, while more resolved phosphate-containing oxide surfaces exhibit *ν*(PO_3_) modes at approximately 1150, 1105, 1036 and 990 cm^−1^, together with a *ν*_S_(P–OH) contribution near 954 cm^−1^.^34,35^ Accordingly, the broad and structured envelopes observed for CoP/Co_2_P@BC and CoP/Co_2_P@BC@AC are assigned to overlapping P–O/PO_*x*_ stretching vibrations from oxidized phosphorus-containing surface species, possible P–O–Co environments, and C–O/C–O–C vibrations from the oxygenated carbon framework.^36^ Owing to the breadth and extensive overlap of these bands, the individual coordination geometry of the phosphate-like species cannot be determined unambiguously from FTIR alone.

Absorptions below approximately 700 cm^−1^ can be tentatively related to Co–O lattice vibrations and low-frequency deformations of P–O–Co or phosphate-like surface species. These features are consistent with the cobalt–oxygen and phosphate-related signatures observed by Raman spectroscopy. Importantly, FTIR does not provide direct identification of Co–P bonds or independently establish the CoP/Co_2_P phases; rather, it supports the presence of oxidized phosphorus-containing species at the composite surface, whereas the phosphide-phase composition is established by the corresponding XPS analysis.

### 3.2. Raman spectroscopy

Raman spectroscopy was employed to explore the vibrational fingerprints of the hybrid architecture in CoP/Co_2_P@BC@AC (Fig. 2b). In the low-wavenumber region, the spectrum exhibits five distinct lattice modes at approximately 196, 474, 518, 602 and 670 cm^−1^, which can be assigned to the F_2g_, E_g_ and A_1g_ phonon symmetries characteristic of cubic Co_3_O_4_. These bands arise from Co–O lattice vibrations associated with Co cations occupying tetrahedral and octahedral sites, indicating that a spinel-type cobalt oxide component is present at or near the catalyst surface, a common outcome of partial surface oxidation during phosphidation and/or post-synthetic handling.^37,38^ In addition, a small contribution in the 900–1050 cm^−1^ window is consistent with the vibrational envelope of phosphate (PO_4_^3−^) species,^39^ suggesting residual phosphate-containing groups or surface phosphate formation within the composite.

At higher wavenumbers, all carbon-containing samples display the characteristic D (∼1350 cm^−1^) and G (∼1580 cm^−1^) bands. The D band arises from defect-activated breathing modes of sp^2^ carbon rings, whereas the G band is associated with the in-plane stretching of sp^2^-bonded carbon. Quantitative analysis yielded *I*_D_/*I*_G_ = 0.885 ± 0.015 and *A*_D_/*A*_G_ = 3.157 ± 0.080 for AC, confirming the presence of a defect-containing sp^2^ carbon framework. For CoP/Co_2_P@BC, these descriptors decrease to *I*_D_/*I*_G_ = 0.826 ± 0.106 and *A*_D_/*A*_G_ = 1.718 ± 0.154, reflecting changes in band shape and spectral weight following incorporation of the cobalt phosphide/oxide phase into the biomass-derived carbon matrix.

The addition of activated carbon to form CoP/Co_2_P@BC@AC produces only a marginal further decrease in the peak-height ratio to *I*_D_/*I*_G_ = 0.809 ± 0.041. Because this difference lies within the experimental uncertainty, the two composites exhibit essentially comparable peak-height D/G responses. The slightly stronger relative G-band contribution in CoP/Co_2_P@BC@AC is consistent with preservation of coherent sp^2^ domains provided by AC. In contrast, its higher integrated ratio, *A*_D_/*A*_G_ = 2.398 ± 0.188, indicates a broader defect-related D-band contribution and increased heterogeneity of the carbon environments within the ternary architecture. Thus, the incorporation of AC modifies primarily the band widths and distribution of sp^2^ carbon environments, rather than producing a pronounced change in the peak-height-derived defect ratio.

### 3.3. Morphological analysis

**Fig. 3:**
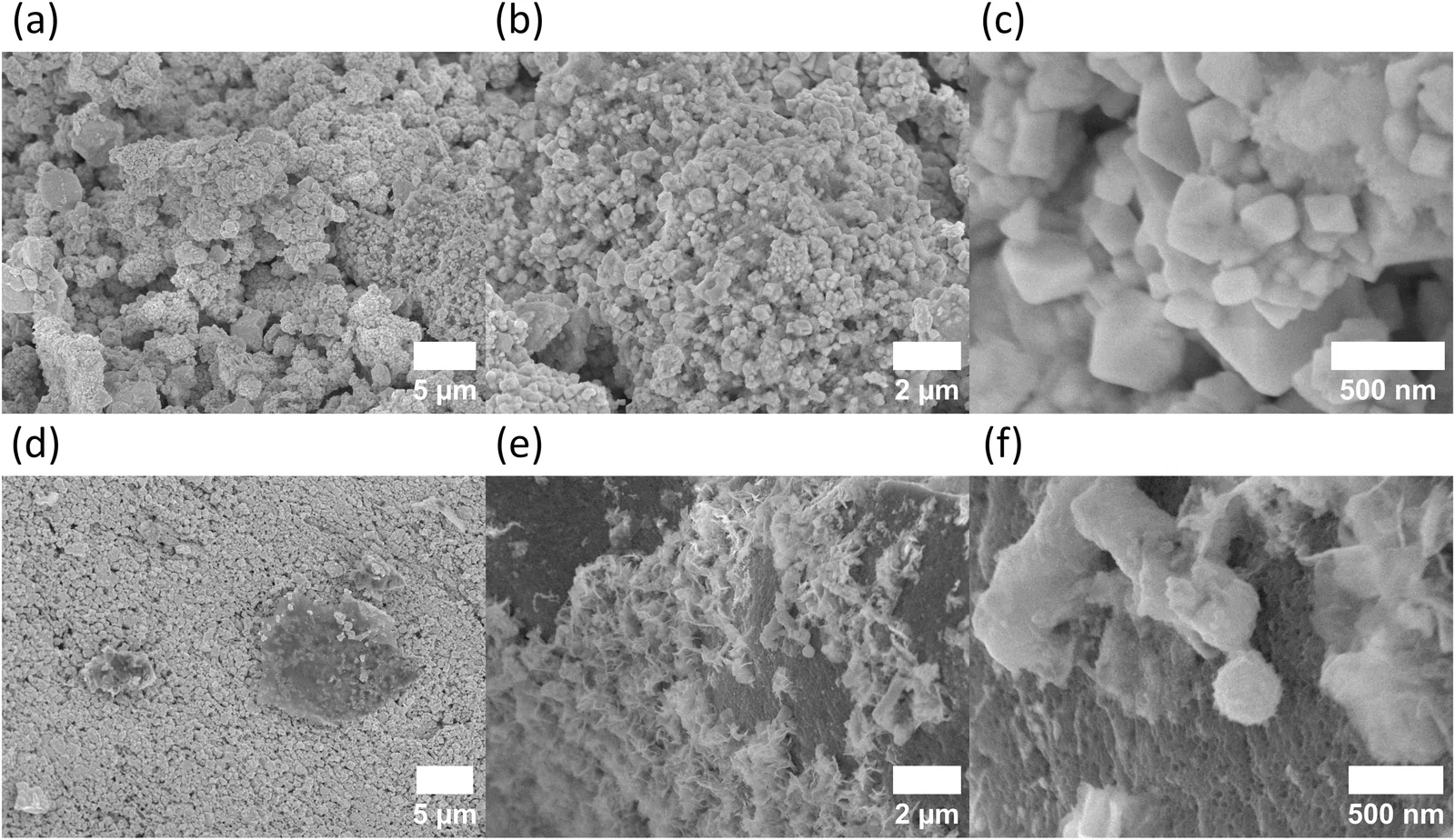
SEM images of CoP/Co_2_P@BC (a–c) and CoP/Co_2_P@BC@AC (d–f).

Scanning electron microscopy (SEM) was used to investigate the morphologies of CoP/Co_2_P@BC and CoP/Co_2_P@BC@AC, as presented in Fig. 3a–f, respectively. CoP/Co_2_P@BC displays a unique morphology, largely composed of cubic particles that exhibit slight agglomeration and relatively large sizes, reflecting the limited morphological control inherent to solid-phase reactions.^40^ In addition, cobalt phosphide particles and minor impurities from the peanut shell extract (*e.g.*, SiO_2_, Al_2_O_3_) are dispersed on the surface and throughout the porous matrix.^37^

Correlated EDS mapping showed that Co, C, N, Si, and Al were uniformly distributed on CoP/Co_2_P (Fig. S1a). CoP/Co_2_P@BC displayed a porous structure, which was further enhanced in CoP/Co_2_P@BC@AC due to the combined effect of biomass-derived carbon (BC) and activated carbon (AC), forming a hierarchically structured, conductive matrix. As illustrated in Fig. S1b, EDS mapping of CoP/Co_2_P@BC@AC confirmed the uniform distribution of Co, P, C, N, Si, and Al throughout the composite. The detected oxygen primarily originates from biomass extracts, precursors, inorganic salts, and oxygen-containing functional groups (*e.g.*, C=O, CO) on the carbon layers.^38^

The porous, hierarchically structured CoP/Co_2_P@BC@AC, with uniformly distributed CoP/Co_2_P nanoparticles, provides abundant exposed active sites and efficient mass transport pathways. These structural advantages, combined with the conductive dual-carbon matrix, underlie the enhanced electrocatalytic performance of CoP/Co_2_P@BC@AC for both HER and OER. Furthermore, the combination of a high specific surface area and porous structure facilitates efficient charge and mass transport, contributing to superior electrocatalytic performance.^39,41^

**Fig. 4:**
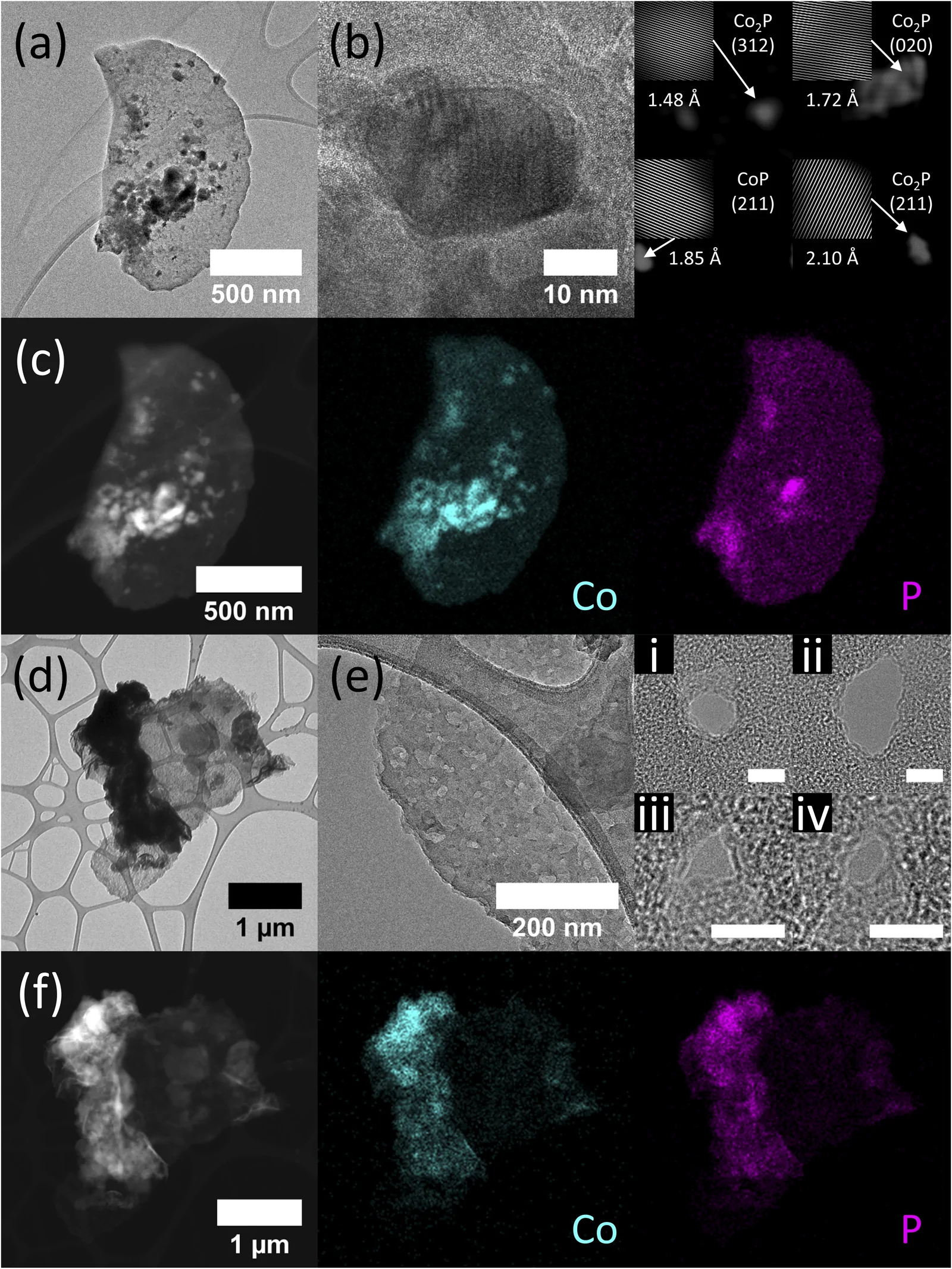
(a) TEM image of CoP/Co_2_P@BC particle. (b) HR-TEM image of BC-supported CoP/Co_2_P nanoparticle with insets showing different lattice planes of CoP and Co_2_P with corresponding interplanar spacing values through four select pairs of fast Fourier transform (FFT) and inverse FFT operations of HR-TEM image. (c) HAADF STEM image and corresponding Co and P EDS elemental maps of CoP/Co_2_P@BC particle. (d) TEM image of CoP/Co_2_P@BC@AC particle. (e) TEM image revealing AC porous fine structure with insets showing HR-TEM images of various pores with different sizes: 5.7 nm (i), 9.9 nm (ii), 2.6 nm (iii), and 2.3 nm (iv), with thin scale bar representing 5 nm. (f) HAADF STEM image and corresponding Co and P EDS elemental maps of CoP/Co_2_P@BC@AC particle.

Fig. 4 shows the (HR-)TEM/HAADF-STEM/EDS characterization of CoP/Co_2_P@BC and CoP/Co_2_P@BC@AC. CoP/Co_2_P NPs appear to populate the BC matrix (Fig. 4a). HR-TEM image (Fig. 4b) and corresponding lattice fringe images of select CoP and Co_2_P lattice planes provide evidence of the coexistence of both phases.^42,43^Fig. 4c shows the HAADF STEM image of the CoP/Co_2_P@BC particle and EDS elemental maps clearly demonstrate homogeneous distributions of Co and P throughout the particle. A particle of CoP/Co_2_P@BC@AC appears in Fig. 4d with relatively more defective surface. The porous nature of CoP/Co_2_P@BC@AC is evident in the presence of various pores with different sizes ranging from 9.9 nm to 2.3 nm (Fig. 4e) confirming successful incorporation of CoP/Co_2_P@BC within AC. Fig. 4f shows the HAADF STEM image of the CoP/Co_2_P@BC@AC particle and EDS elemental maps demonstrate distributions of Co and P that are relatively more homogeneous than those of CoP/Co_2_P@BC reflecting another role for AC incorporation in fine distribution of NPs throughout the matrix.

### 3.4. XPS

Elemental quantification was made possible by survey wide-scan XPS, as shown in Fig. S2 and summarized in Table S1. The atomic ratio of cobalt to phosphorus (Co : P) changes from 0.92 : 1 for CoP/Co_2_P@BC to 0.22 : 1 for CoP/Co_2_P@BC@AC. High-resolution XPS analysis revealed a significant variation in chemical environments experienced by the atomic constituents of CoP/Co_2_P@BC and CoP/Co_2_P@BC@AC. The variation in the surface Co : P ratio (0.92 : 1–0.22 : 1) arises from the surface-sensitive nature of XPS and likely reflects phosphorus enrichment due to surface oxidation and segregation, which can impact the distribution of catalytically active sites.

**Fig. 5:**
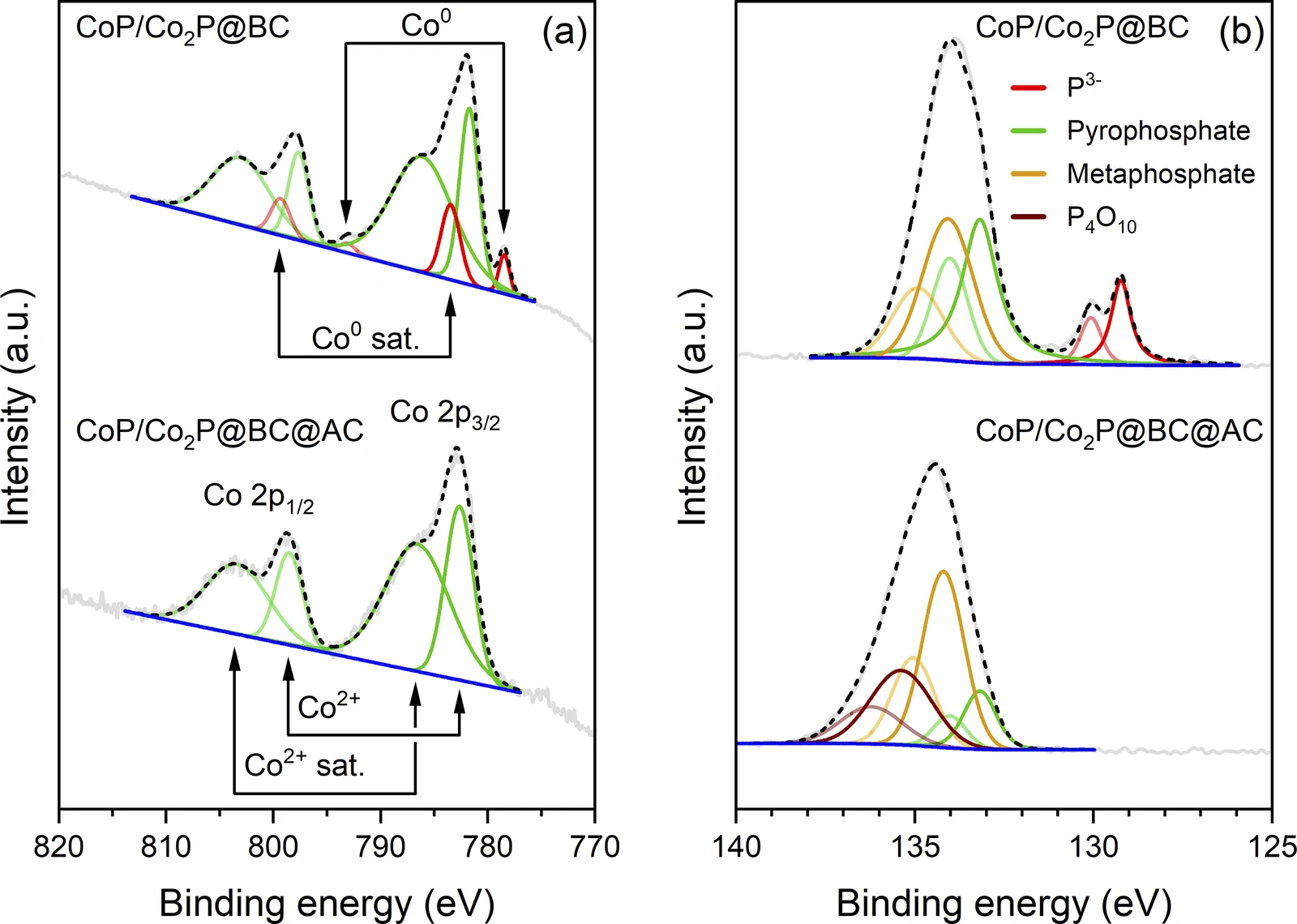
Analyzed XP Co 2p (a) and P 2p (b) spectra of CoP/Co_2_P@BC and CoP/Co_2_P@BC@AC.

Table S2 provides a summary of the XPS analysis of C 1s, O 1s, N 1s, P 2p, and Co 2p. Fig. S3 shows the analyzed C 1s, O 1s, and N 1s XP spectra. C 1s spectra were deconvoluted to six components: C=C, C–C, C–O, C=O, COO, and π–π*.^44–46^ K 2p contribution appears in both samples.^44–47^ O 1s spectra were deconvoluted to five components: M–O, C=O, C–O, COO, and O_2_/CO_2_.^47^Fig. 5 shows the analyzed Co 2p and P 2p XP spectra. Co 2p region was fit using up to eight components (binding energy for Co 2p_3/2_ in eV): Co^0^ spin–orbit doublet (778.5 eV), Co^2+^ spin–orbit doublet (781.7 eV), Co^0^ satellite spin–orbit doublet (783.5 eV), and Co^2+^ satellite spin–orbit doublet (786.1 eV).^47^ Co^0^ and Co^2+^ components discernibly differ in magnitude of the spin–orbit splitting: 14.61 eV and 15.93 eV, respectively. Co^0^ and Co^2+^ exhibit distinct effective nuclear charges (*Z*_eff_) and this influences the spin–orbit coupling; a higher *Z*_eff_ for Co^2+^ induces stronger spin–orbit coupling which is reflected by more pronounced splitting. Analysis confirms the presence of 14 at% metallic Co (Co^0^) and 86 at% Co^2+^ in CoP/Co_2_P@BC and presence of Co^2+^ solely in CoP/Co_2_P@BC@AC. P 2p XPS analysis evidences presence of P^3−^, pyrophosphate, metaphosphate, and P_4_O_10_.^47^ In CoP/Co_2_P@BC, P makes up 14 at% P^3−^, 43 at% pyrophosphate, 42 at% metaphosphate. In CoP/Co_2_P@BC@AC, P makes up 15 at% pyrophosphate, 52 at% metaphosphate, and 33 at% P_4_O_10_. It means that the Co and P have partial positive charges (*δ*^+^) and negative charges (*δ*^−^), respectively, indicating that the electron density transfers from Co to P.^41,48^ From previous studies, this significant electronic modulation and CoP/Co_2_P heterostructure is beneficial to facilitate the electrocatalytic activity.^49–51^ Indeed, the observed changes in atomic structure reflect the impact of incorporating activated carbon, enhancing the dispersion of active phases, increasing the accessibility of catalytic sites, and facilitating both electronic and ionic transport within the porous network.^52,53^

### 3.5. Electrochemical activity

The electrocatalytic OER and HER performances of the as-synthesized CoP/Co_2_P@BC and CoP/Co_2_P@BC@AC materials (catalyst loading: 82 µg cm^−2^) were evaluated using nickel foam as the working electrode, in Ar-saturated 1 M KOH electrolyte. Measurements were performed in a standard three-electrode setup at scan rates of 100 mV s^−1^ and 50 mV s^−1^, with all potentials referenced to the reversible hydrogen electrode (RHE).

**Fig. 6:**
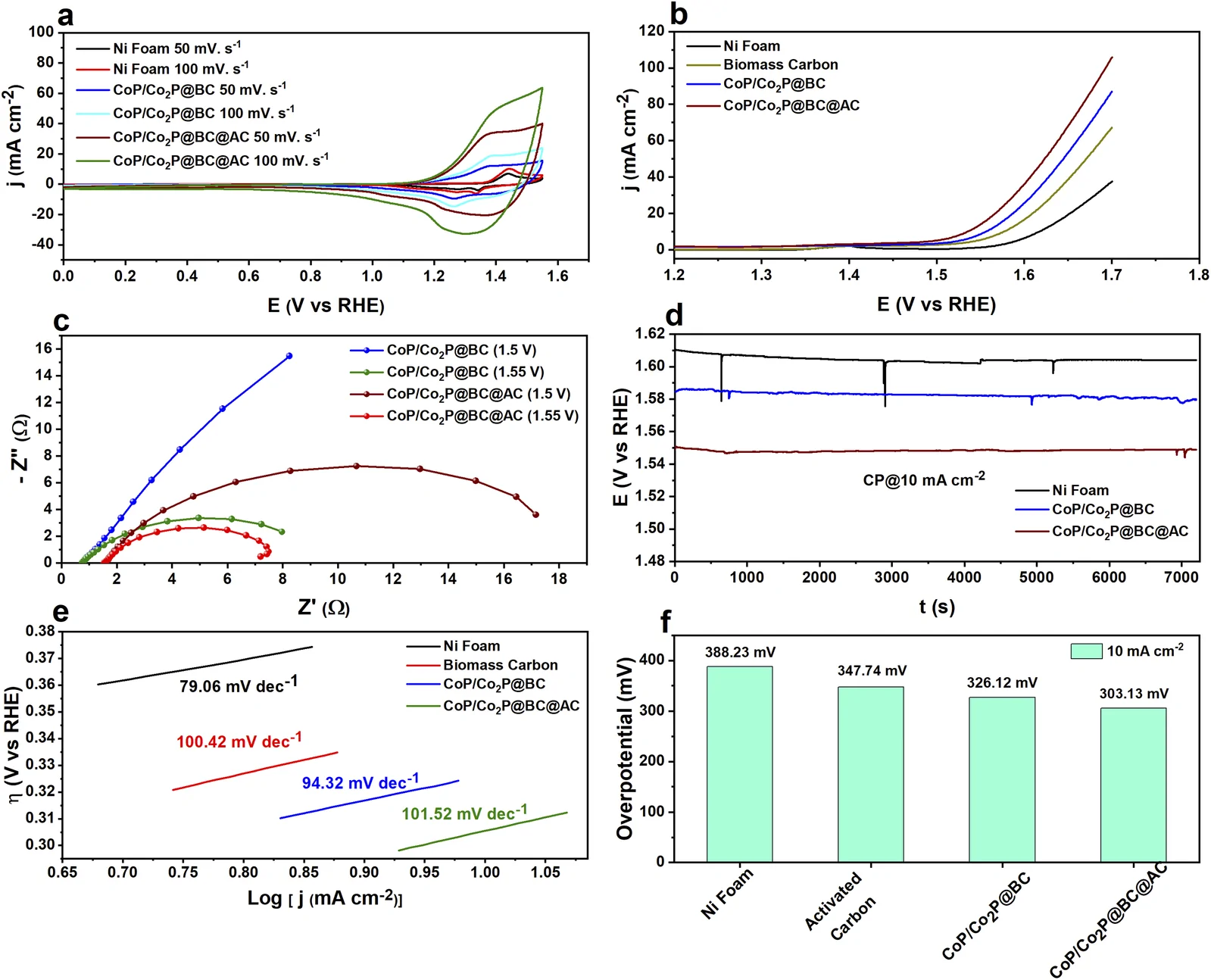
OER performances of CoP/Co_2_P@BC and CoP/Co_2_P@BC@AC samples in 1 M KOH solution. (a) Cyclic voltammetry. (b) Linear sweep voltammetry. (c) EIS curve. (d) Chronopotentiometry curves. (e) Tafel slope. (f) Overpotentials at current density of 10 mA cm^−2^.

**Fig. 7:**
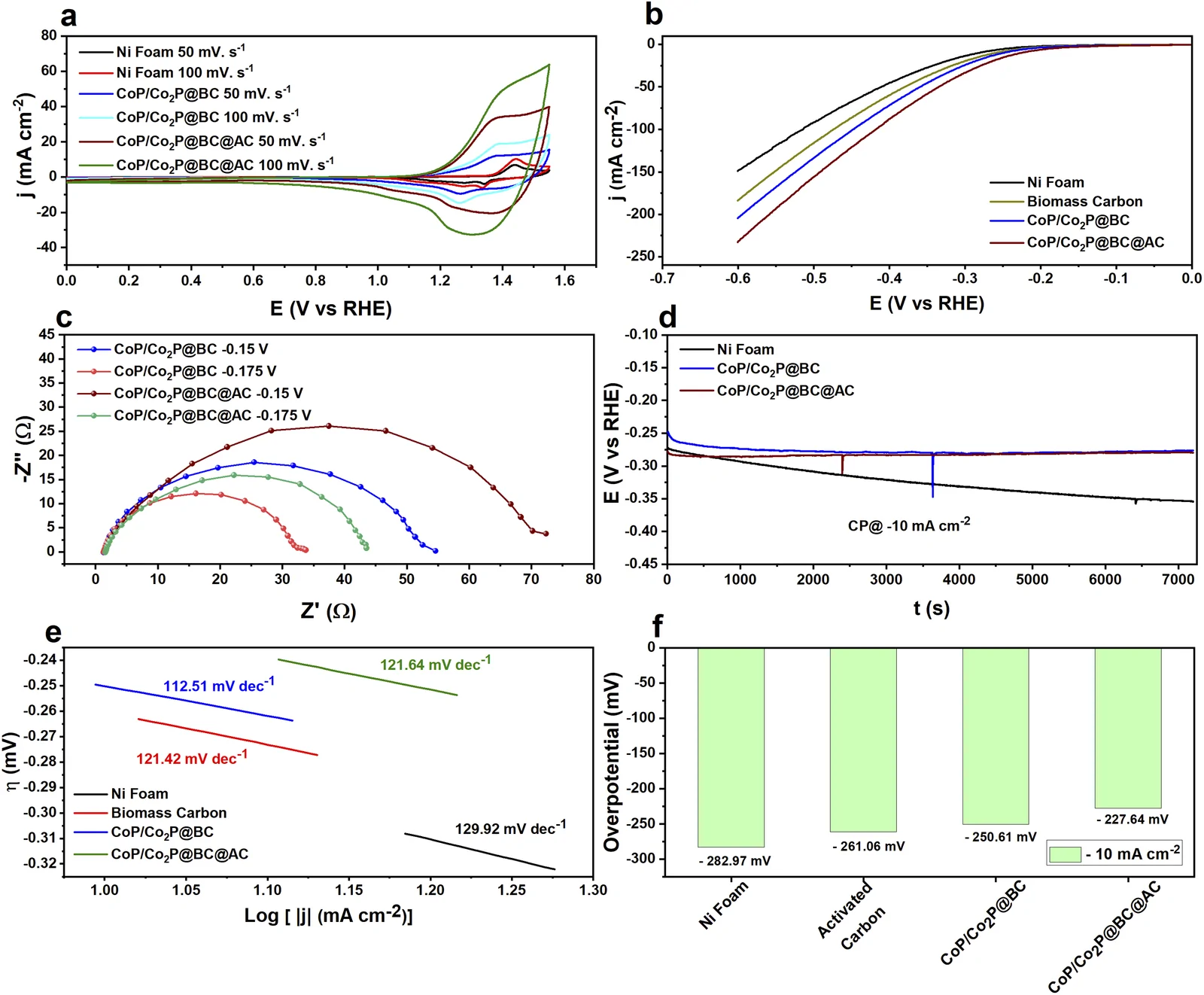
HER performances of CoP/Co_2_P@BC and CoP/Co_2_P@BC@AC samples in 1 M KOH solution. (a) Cyclic voltammetry. (b) Linear sweep voltammetry. (c) EIS curve. (d) Chronopotentiometry curves. (e) Tafel slope. (f) Overpotentials at current density of 10 mA cm^−2^.

Accordingly, the OER and HER performances of all electrodes were first examined by CV in Ar-saturated 1.0 M KOH, at scan rates of 100 mV s^−1^ and 50 mV s^−1^ respectively (Fig. 6a and 7a), over a potential range of 0.0–1.55 V *vs.* RHE. In these measurements, the Ni^2+^/Ni^3+^ oxidation is observed between 1.4 and 1.5 V *vs.* RHE, whereas the onset of OER occurs at approximately 1.55 V *vs.* RHE.

On the reverse scan, the Ni^3+^/Ni^2+^ reduction is observed between 1.3 and 1.4 V *vs.* RHE for the Ni foam.^54^ Linear sweep voltammetry (LSV) curves were recorded at a scan rate of 5 mV s^−1^ as shown in Fig. 6b and 7b.

The CoP/Co_2_P@BC@AC composite required an overpotential of only 0.303 V to reach 10 mA cm^−2^, lower than that of CoP/Co_2_P@BC (0.326 V) (Fig. 6b). This lower overpotential indicates superior OER activity, mainly due to the physicochemical properties of metallic Co (electronic and geometric structure) and the carbon active sites induced by N and S doping.^38,55^ We used electrochemical impedance spectroscopy (EIS) to further investigate the electrode process kinetics under the catalytic OER operating conditions.

As shown in Fig. 6c, the Nyquist plots reveal distinct semicircular profiles corresponding to the charge transfer resistance (*R*_ct_) of the electrodes.

Among all samples, CoP/Co_2_P@BC@AC (1.55 V) exhibits the smallest semicircle, indicating the lowest charge-transfer resistance (*R*_ct_) and the most efficient charge-transfer kinetics during the oxygen evolution reaction (OER). This reduced *R*_ct_ reflects more efficient electron transport across the electrode–electrolyte interface.^38^

In contrast, CoP/Co_2_P@BC (1.5 V) displays the largest semicircle, suggesting a higher interfacial resistance. However, the remarkable decrease in *R*_ct_ for the CoP/Co_2_P@BC@AC catalyst shows that adding activated carbon significantly boosts electrical conductivity, speeds up ion and electron transport, and promotes charge transfer at the interface.

Furthermore, the synergistic interaction between the CoP and Co_2_P phases, combined with the conductive porous carbon matrix, contributes to the superior electrocatalytic performance of the CoP/Co_2_P@BC@AC system.^56^

Further, the long-term stability of the catalyst was tested using the chronopotentiometric measurement at a constant current density of *j* = 10 mA cm^−2^ in OER for 2 h (Fig. 6d). However, the CoP/Co_2_P@BC@AC composite electrolyzer exhibited high stability with only a small degradation of 8.7% of the initial current density over 2 h under the same applied cell potential.

As shown in Fig. 6e, the CoP/Co_2_P@BC@AC and CoP/Co_2_P@BC catalysts display OER Tafel slopes of 101.52 mV dec^−1^ and 94.32 mV dec^−1^, respectively, indicating moderately slow charge-transfer kinetics and a multistep electron-transfer mechanism. The good OER activity of the CoP/Co_2_P@BC@AC composite likely comes from the added carbon, which provides a large specific surface area and helps with rapid electron transfer.

Analogous behavior was observed for the HER performance of CoP/Co_2_P@BC and CoP/Co_2_P@BC@AC in 1 M KOH. Among the prepared electrocatalysts, CoP/Co_2_P@BC@AC showed a lower overpotential of −227 mV, compared to of CoP/Co_2_P@BC composite (−250 mV) at the high current density of 10 mA cm^−2^ (Fig. 7b).

Besides, Fig. 7c shows the Electrochemical Impedance Spectroscopy (EIS) Nyquist plots of the CoP/Co_2_P@BC and CoP/Co_2_P@BC@AC electrodes, measured under hydrogen evolution reaction (HER) conditions at applied potentials of −0.250 V and −0.275 V. These Nyquist plots reveal that the CoP/Co_2_P@BC@AC catalyst exhibits significantly reduced charge-transfer resistance compared to CoP/Co_2_P@BC, particularly at −0.275 V.

This result demonstrates that the introduction of activated carbon improves electrical conductivity and enhances the interfacial charge transport kinetics. Moreover, the synergy between CoP and Co_2_P phases provides abundant electroactive sites for hydrogen evolution, accelerating reaction kinetics and improving the HER performance of the CoP/Co_2_P@BC@AC electrodes.^56–59^

Further, the long-term stability of the best electrolyzer, CoP/Co_2_P@BC and CoP/Co_2_P@BC@AC composite was also evaluated using the chronopotentiometric measurement at a constant current density of *j* = −10 mA cm^−2^ for 2 h.

As shown in Fig. 7d, CoP/Co_2_P@BC@AC composite and CoP/Co_2_P@BC show good stability during HER catalysis with only a small degradation of 5.76% and 10.82% respectively, of the initial current density over 2 h under the same applied cell potential.

The CoP/Co_2_P@BC@AC catalyst exhibited a Tafel slope of 121.64 mV dec^−1^. This value differs from that of the CoP/Co_2_P@BC catalyst, which showed a Tafel slope of 112.51 mV dec^−1^ (Fig. 7e). This Tafel slope of approximately 120 mV dec^−1^ indicates that the HER proceeds *via* the Volmer–Heyrovsky mechanism, with the Volmer step (electrochemical proton adsorption) as the rate-determining step.

This suggests relatively sluggish proton discharge kinetics, while the conductive and porous BC@AC framework helps facilitate charge and mass transport, partially enhancing the overall HER performance.^60^

In summary, the superior electrocatalytic performance of CoP/Co_2_P@BC@AC can be attributed to the synergistic interplay between the CoP and Co_2_P phases and the porous carbon matrix. CoP, with its covalent Co–P bonds and partially filled Co 3d orbitals, provides moderate binding sites for reaction intermediates and excellent electrical conductivity, facilitating rapid electron transfer. Co_2_P, being more metallic, complements CoP by offering additional active sites and enhancing charge transport.^58^

During the OER process, partial surface oxidation of CoP and Co_2_P leads to the formation of Co–P–O and CoOOH species, which serve as the actual active sites, promoting OH^−^ adsorption and accelerating reaction kinetics.^61^

The high surface area and porosity of the BC@AC support further enhance mass transport of reactants and products, while the graphitic carbon framework ensures effective electron conduction and prevents aggregation of the phosphide nanoparticles.^62^

Together, these factors create a heterogeneous surface that optimizes intermediate adsorption and facilitates rapid charge transfer, ultimately improving catalytic efficiency for both HER and OER. This shows how important phase composition and carbon architecture are in tuning electrochemical activity.^58^

Therefore, to achieve the best HER performance, it is crucial to use thicker layers of carbon and porous carbon.

Then, the proper carbon layer is significant in the balance of activity with stability and is a requirement that needs to be considered in the preparation of electrocatalysts.^63^

Further, a porous carbon matrix (PCM) with high conductivity, good stability, and confined microenvironment is considered as an ideal support to stabilize isolated metal centers for electrocatalysis.^64,65^

The use of peanut shell extract as a precursor for the CoP/Co_2_P@BC synthesis showed satisfactory catalytic activity for both half-reactions. This performance comes from carbon layers doped with different elements, derived from peanut shell extract that coats the CoP. These layers show strong catalytic activity for the HER and help the carbon turn from amorphous to graphitized during heating.^63,65,66^

Therefore, the graphitic carbons may provide good conductivity, which will facilitate the electron transfer on the electrode and thus it is beneficial for the electrocatalytic reactions.^67^ Carbon-encapsulated HER electrocatalysts are promising for water-splitting applications for three main reasons: they enhance conductivity, optimize the hydrogen adsorption energy (Δ*G*_H*_) values on the material's surface, and improve corrosion resistance.^63^

In addition, the synergy between CoP and Co_2_P likely lowers the Δ*G*_H*_ and causes conduction band bending. Meanwhile, the peanut shell-derived carbon provides a large specific surface area and good electron-donating properties.^38^

Table 1 presents a comparative analysis of the electrocatalytic performance of biomass-derived electrocatalysts and the catalyst developed in this work. Compared to other CoP/Co_2_P-based catalysts reported so far, this work is unique because it combines a truly sustainable carbon structure made from two biomass sources with a well-defined CoP/Co_2_P heterostructure. Made through a simple and scalable method, it delivers balanced HER/OER activity, low charge-transfer resistance, and excellent durability.

**Table 1:** Comparative performance of biomass-derived electrocatalysts in 1 M KOH at 10 mA cm^−2^

Electrocatalyst	HER performance	OER performance	Reference
Co_2_P@NPPC	*η* = 147 mV	*η* = 316 mV	68
CoP/Co_2_P@ACNF-800, on sugarcane-derived carbon	*η* = 155 mV	*η* = 291 mV	41
CoP–Co_2_P/coal-based carbon fibers (CoP–Co_2_P/C-CFs)	*η* = 115 mV	*η* = 247 mV	69
CoP/Co_2_P loaded on cellulose nanofiber-derived carbon aerogels delivered	*η* = 63 mV	*η* = 277 mV	70
Review of biomass-based carbons	*η* > 100 mV	OER overpotentials variable	71
	Tafel slopes		
	50–150 mV dec^−1^		
CoP/Co_2_P@BC@AC	*η* = 227 mV	*η* = 303 mV	This work
	121 mV dec^−1^	101 mV dec^−1^	

## 4 Conclusion

In summary, this work successfully reported the synthesis of heterostructure CoP/Co_2_P@BC and CoP/Co_2_P@BC@AC composites as bifunctional catalysts for electrochemical water splitting by hydrothermal treatment followed by phosphidation, in which peanut shell extract and activated carbon derived from peanut shell were used as the precursors.

The synthesized electrocatalysts presented outstanding catalytic activity in alkaline medium for OER, with a low overpotential of 303 mV for CoP/Co_2_P@BC@AC composite which is superior to CoP/Co_2_P@BC, which exhibited 326 mV, at a current density of 10 mA cm^−2^. However, in HER reaction, CoP/Co_2_P@BC@AC composite has exhibited low overpotential of 227 mV unlike CoP/Co_2_P@BC, which exhibited an overpotential of 250 mV, at a current density of −10 mA cm^−2^.

This study showed that the use of peanut shell extract poses as an alternative low-cost carbon source favorable to electrocatalytic activity. However, this result could call into question the nature of the activated carbon encapsulated in the catalytic materials for the HER and OER reactions.

This work offers new ideas for future sustainable energy conversion devices based on inexpensive and efficient non-precious metals and biomass-derived graphitic carbon.

## Conflicts of interest

The authors declare no conflict of interest.

## Supplementary Material

RA-016-D6RA05091C-s001

## Data Availability

The data supporting the findings of this study are available within the article and its supplementary information (SI). Additional data related to this work are available from the corresponding author upon reasonable request. Supplementary information is available. See DOI: https://doi.org/10.1039/d6ra05091c.
