# Supplementary material for: CoP/Co2P heterostructures embedded in biomass-derived carbons as efficient electrocatalysts for hydrogen and oxygen evolution reactions
Source: RSC Adv. 2026 Aug 7;16(40):46134–45. doi: 10.1039/d6ra05091c (PMC13448766; doi:10.1039/d6ra05091c)
Supplement: Supplementary file 1 [file RA-016-D6RA05091C-s001.pdf]

Supplementary Information (SI) for RSC Advances.
This journal is © The Royal Society of Chemistry 2026

Supplementary Information

CoP/Co_2P Heterostructure Embedded in Biomass-Derived Carbons as Efficient Electrocatalysts for Hydrogen and Oxygen Evolution Reactions

Abdourahmane DIENG[1], Ahmed SUBRATI[2], Zahra HAGHEH-KAVOUSI[3], Ndeye M Ndiaye[1], Yaovi HOLADE[3], Eduart Gutierrez-Pineda[2], Sergio E. Moya[2], Balla D NGOM*[1]

[1] Laboratoire de Photonique Quantique d'Energie & NanoFabrication (LPQEN), Faculté des Sciences et Techniques, Département de Physique, Université Cheikh Anta Diop de Dakar, BP5005 Dakar-Fann, Dakar, Sénégal.

[2] Center for Cooperative Research in Biomaterials (CIC biomaGUNE), Basque Research and Technology Alliance (BRTA), San Sebastian 20014, Spain

[3] Institut Européen des Membranes, IEM, UMR, 5635, Univ Montpellier, ENSCM, CNRS, 34090 Montpellier, France.

***Corresponding author**

E-mail: bdngom@gmail.com; Tel: +221 77 441 66 44

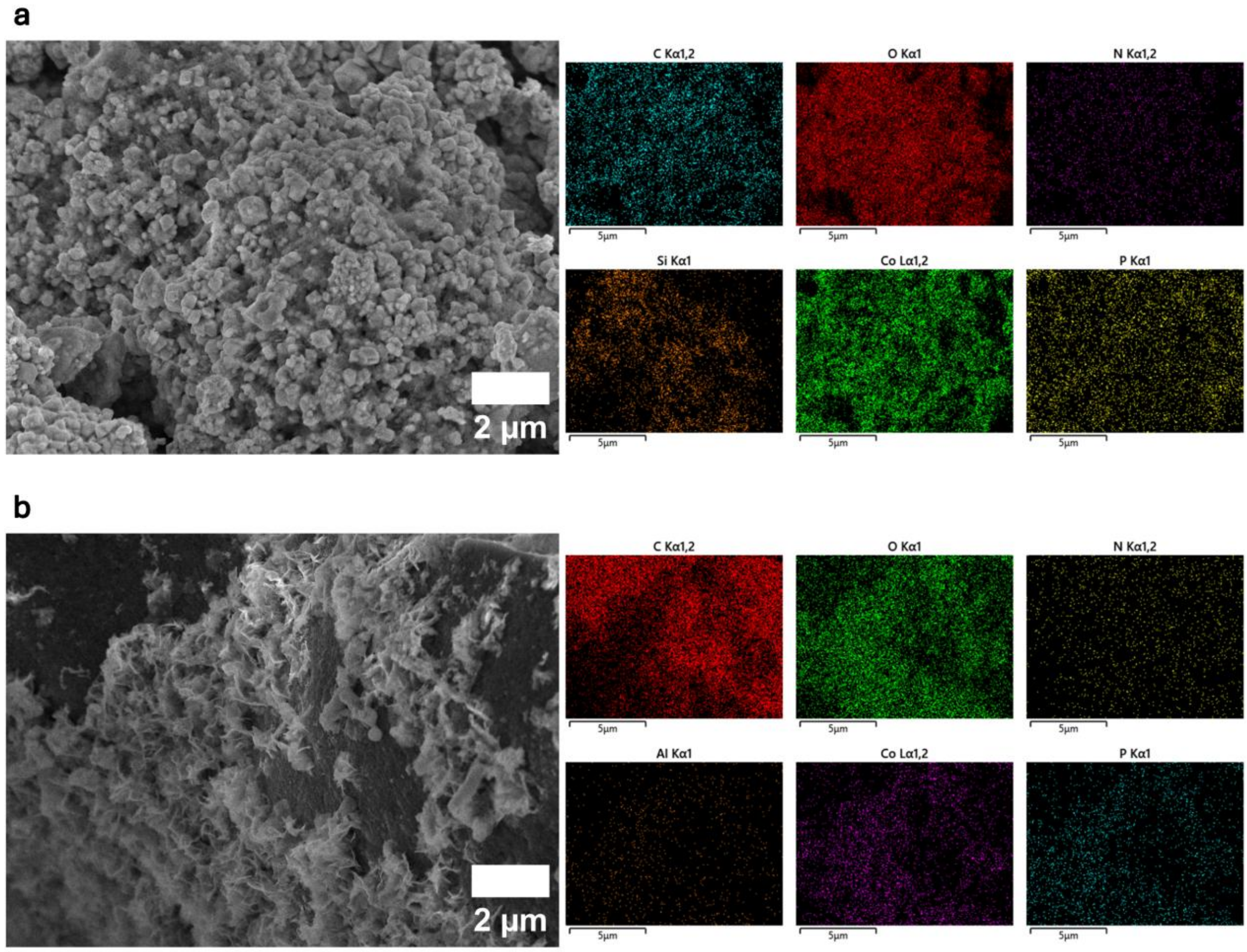

Figure S1. SEM/EDS elemental mapping of (a) CoP/Co_2P@BC and (b) CoP/Co_2P@BC@AC.

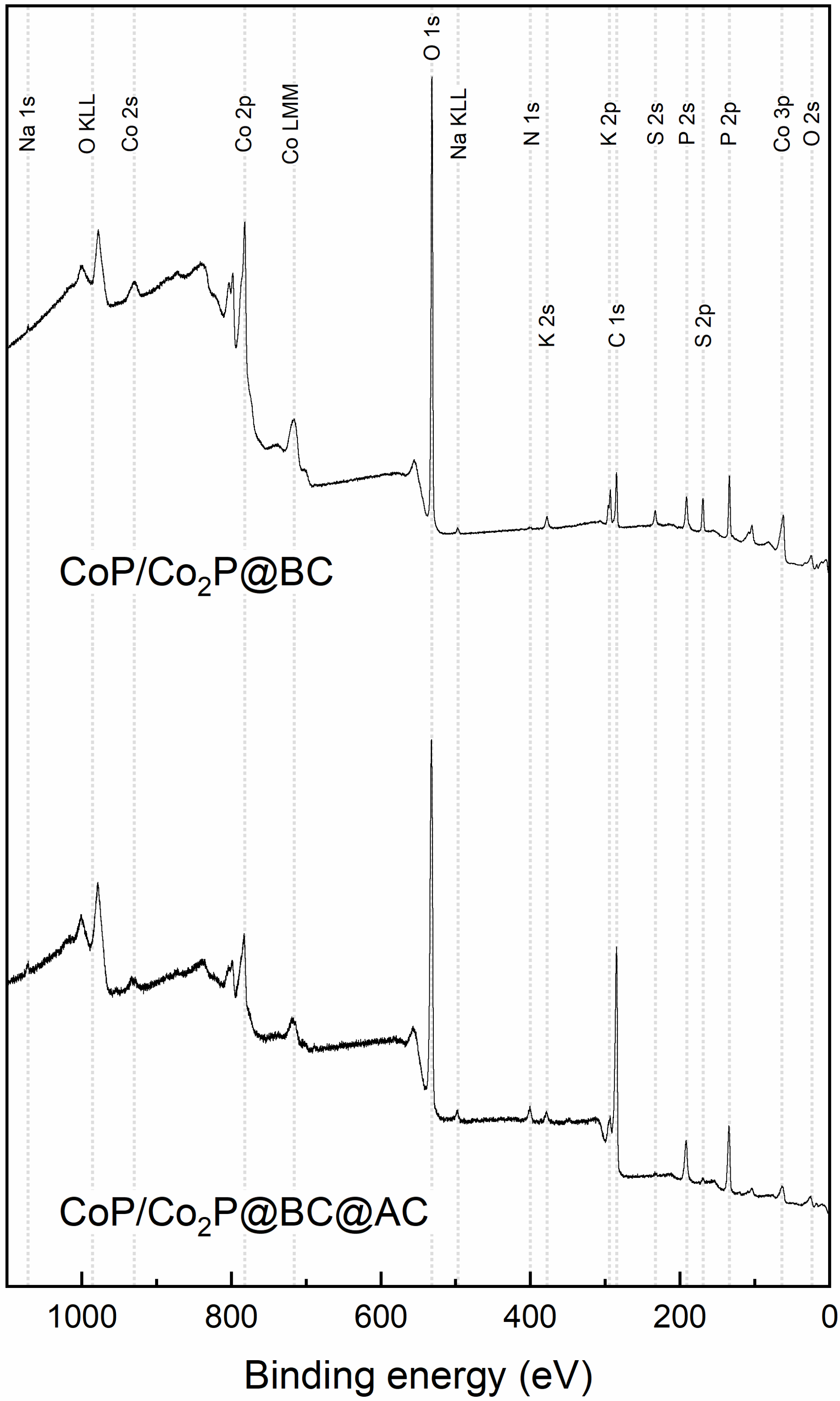

Figure S2. Wide-scan (survey) XP spectra of $CoP/Co_2P@BC$ and $CoP/Co_2P@BC@AC$.

Table S1. Elemental composition *via* survey (wide-scan) XPS.

Sample	Al	C	Co	K	N	Na	O	P	S
CoP/Co_2P@BC	0.00	17.50	13.14	3.11	0.45	0.17	45.89	14.27	5.47
CoP/Co_2P@BC@AC	0.57	50.53	2.50	1.55	2.41	0.25	30.58	11.18	0.43

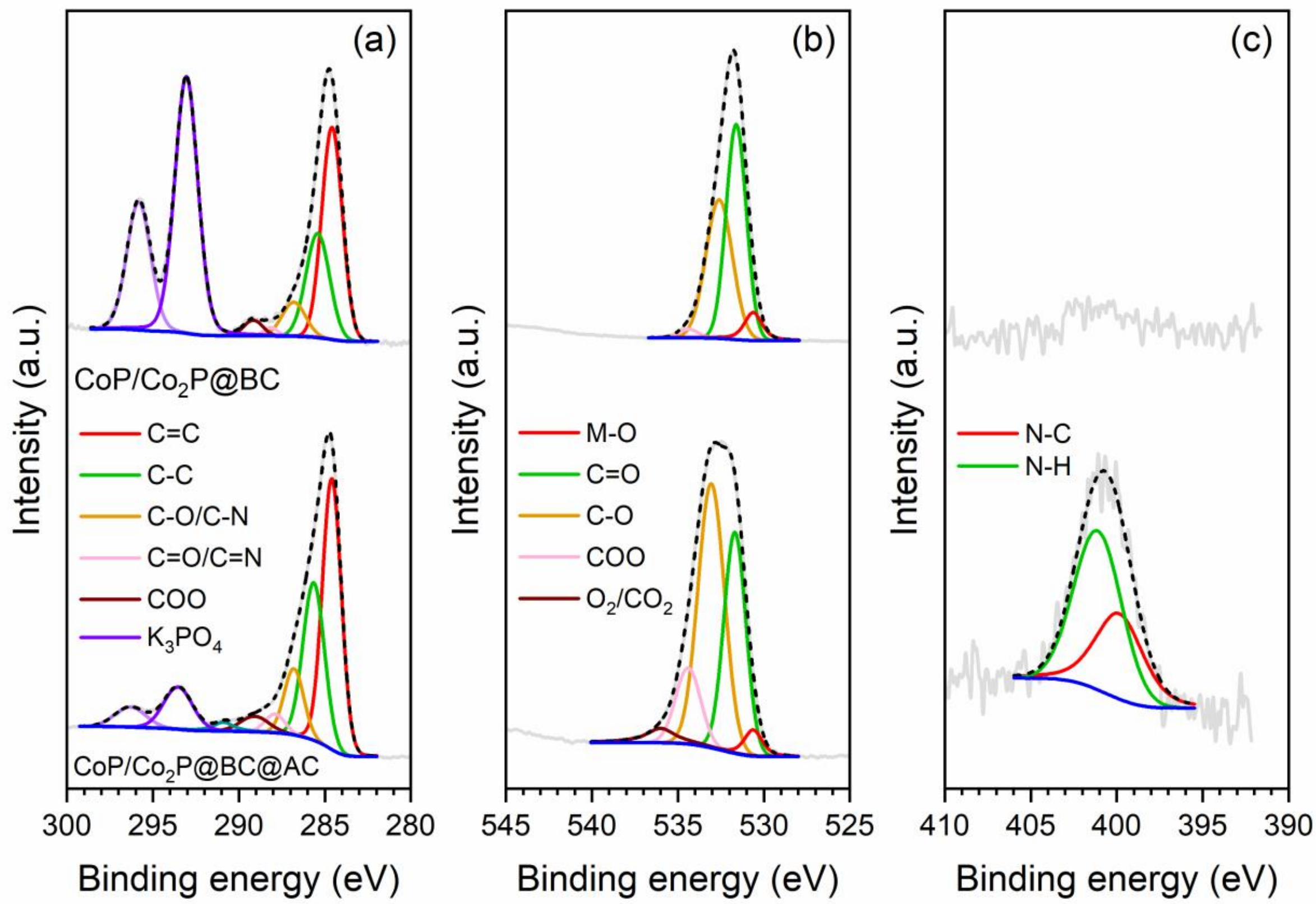

Figure S3. C 1s (a), O 1s (b), and N 1s (c) XP spectra of CoP/Co_2P@BC and CoP/Co_2P@BC@AC.

Table S2. XPS analysis of CoP/Co_2P@BC and CoP/Co_2P@BC@AC.

CoP/Co_2P@BC	C 1s	Component	C=C	C-C	C-O/C-N	C=O/C=N	COO	π- π*	K_3PO_4 K $2p_{3/2}$	K_3PO_4 K $2p_{1/2}$
		B.E. (eV)	284.6	285.4	286.8	288.2	289.2	-	293.1	295.8
		At.%	25.0	14.1	4.5	0.7	1.7	-	36.0	18.0
	O 1s	Component	C-O	C=O	COO	M-O				
		B.E. (eV)	532.6	531.6	534.4	530.6				
		At.%	41.7	49.2	2.0	7.1				
	P 2p	Component	P^{3-} $2p_{3/2}$	P^{3-} $2p_{1/2}$	Pyrophosphate $2p_{3/2}$	Pyrophosphate $2p_{1/2}$	Metaphosphate $2p_{3/2}$	Metaphosphate $2p_{1/2}$	P_4O_{10} $2p_{3/2}$	P_4O_{10} $2p_{1/2}$
		B.E. (eV)	129.2	130.1	133.2	134.0	134.1	134.9	-	-
		At.%	9.4	4.7	28.9	14.5	28.3	14.2	-	-
	Co 2p	Component	Co^0 $2p_{3/2}$	Co^0 $2p_{1/2}$	Co^{2+} $2p_{3/2}$	Co^{2+} $2p_{1/2}$	Co^0 sat. $2p_{3/2}$	Co^0 sat. $2p_{1/2}$	Co^{2+} sat. $2p_{3/2}$	Co^{2+} sat. $2p_{1/2}$
		B.E. (eV)	778.5	793.1	781.7	797.7	783.5	799.3	786.1	803.1
		At.%	2.1	1.0	18.1	9.0	7.2	3.6	39.3	19.7
CoP/Co_2P@BC@AC	C 1s	Component	C=C	C-C	C-O/C-N	C=O/C=N	COO	π- π*	K_3PO_4 K $2p_{3/2}$	K_3PO_4 K $2p_{1/2}$
		B.E. (eV)	284.6	285.6	286.8	287.9	289.1	290.9	293.5	296.3
		At.%	40.6	26.4	10.1	2.9	3.5	1.9	9.7	4.8
	O 1s	Component	C-O	C=O	COO	M-O	O_2/CO_2			
		B.E. (eV)	533.1	531.7	534.4	530.6	536.0			
		At.%	46.6	32.5	12.8	3.9	4.2			
	N 1s	Component	N-C	N-H						
		B.E. (eV)	399.8	401.1						
		At.%	38.4	61.6						
	P 2p	Component	P^{3-} $2p_{3/2}$	P^{3-} $2p_{1/2}$	Pyrophosphate $2p_{3/2}$	Pyrophosphate $2p_{1/2}$	Metaphosphate $2p_{3/2}$	Metaphosphate $2p_{1/2}$	P_4O_{10} $2p_{3/2}$	P_4O_{10} $2p_{1/2}$
		B.E. (eV)	-	-	133.2	134.0	134.2	135.0	135.4	136.2
		At.%	-	-	10.0	5.0	35.0	17.5	21.7	10.9
	Co 2p	Component	Co^{2+} $2p_{3/2}$	Co^{2+} $2p_{1/2}$	Co^{2+} sat. $2p_{3/2}$	Co^{2+} sat. $2p_{1/2}$				
		B.E. (eV)	782.6	798.5	786.6	803.4				
		At.%	24.2	12.1	42.4	21.2				
